# Unilateral Thyroid Lobe Involvement of Graves Disease

**DOI:** 10.1210/jcemcr/luad023

**Published:** 2023-03-06

**Authors:** Yazan Alzedaneen, Meaghan Chelsea Moxley, Rashmi Reddy, Kashif M Munir

**Affiliations:** Internal Medicine Department, University of Maryland Medical Center, Baltimore, MD 21201, USA; Department of Endocrinology, University of Maryland Upper Chesapeake Health - Endocrinology Association, Aberdeen, MD 21001, USA; Department of Endocrinology, Evergreen Health Diabetes and Endocrinology, Kirkland, WA 98034, USA; Division of Endocrinology, Diabetes, and Nutrition, Department of Medicine, University of Maryland School of Medicine, Baltimore, MD 21201, USA

**Keywords:** unilateral goiter, Graves disease, hyperthyroidism, graves orbitopathy

## Abstract

A 30-year-old man presented with 3-year history of Graves disease. He was initially diagnosed after he developed unilateral proptosis and was initiated on methimazole 5 mg, on which he was currently euthyroid. Visible right-sided thyromegaly and trouble swallowing developed 2 months after presentation to our practice. Biochemical evaluation revealed suppressed TSH, normal free T4 and total T3, and elevated thyroid stimulating immunoglobulin with normal thyroid receptor antibody. An ultrasound of the thyroid demonstrated left-sided small nodules with right-sided thyromegaly. A nuclear medicine uptake scan revealed significantly greater uptake in the right thyroid lobe, with overall minimal uptake in the left lobe. The need for definitive therapy that would not exacerbate orbitopathy was discussed, and the patient elected for a right-sided hemithyroidectomy. Postoperative biochemical evaluation demonstrated biochemical euthyroidism despite continued elevation in thyroid stimulating immunoglobulin and newly elevated thyroid receptor antibody while remaining off methimazole. Graves disease can rarely involve a single thyroid lobe. Given the rarity, further investigation is needed to determine the natural course of this form of Graves disease.

Graves disease is an autoimmune disease involving the thyroid gland that is characterized by diffuse goiter, thyrotoxicosis, and infiltrative orbitopathy. The pathophysiology includes the stimulatory action of IgG against TSH receptors, which results in diffuse goiter and unregulated release of thyroid hormones with thyrotoxic symptoms. Unilateral involvement of the thyroid gland is an uncommon, poorly studied presentation of Graves disease. Here, we report a case 30-year-old man who presented with unilateral thyromegaly and unilateral uptake on I-131 scan. This presentation has been infrequently reported in the literature with approximately 20 cases reported worldwide.

## Case Presentation

A 30-year-old man presented with a history of Graves disease that was being treated with methimazole 5 mg daily. Three years before presentation, new-onset proptosis initially led to testing confirming hyperthyroidism from Graves disease. Initial management was with methimazole and propranolol. Beta-blockade was discontinued after several months when thyroid function tests returned to normal. Methimazole dose was tapered to 5 mg daily with normal thyroid function. However, 2 months later, symptoms of visible right-sided thyromegaly and trouble swallowing appeared. He was not experiencing any palpitations, anxiety, trouble sleeping, weight changes, tremors, or diaphoresis. He also denied any eye pain, tearing, or grittiness (clinical activity score = 1). A physical examination showed right eye proptosis with redness in both eyes that was worse in the right but otherwise with no tearing. There was also right-sided thyromegaly with no palpable nodules or lymphadenopathy. There was no tachycardia, diaphoresis, or tremors observed.

## Diagnostic Assessment

**Figure 1.**
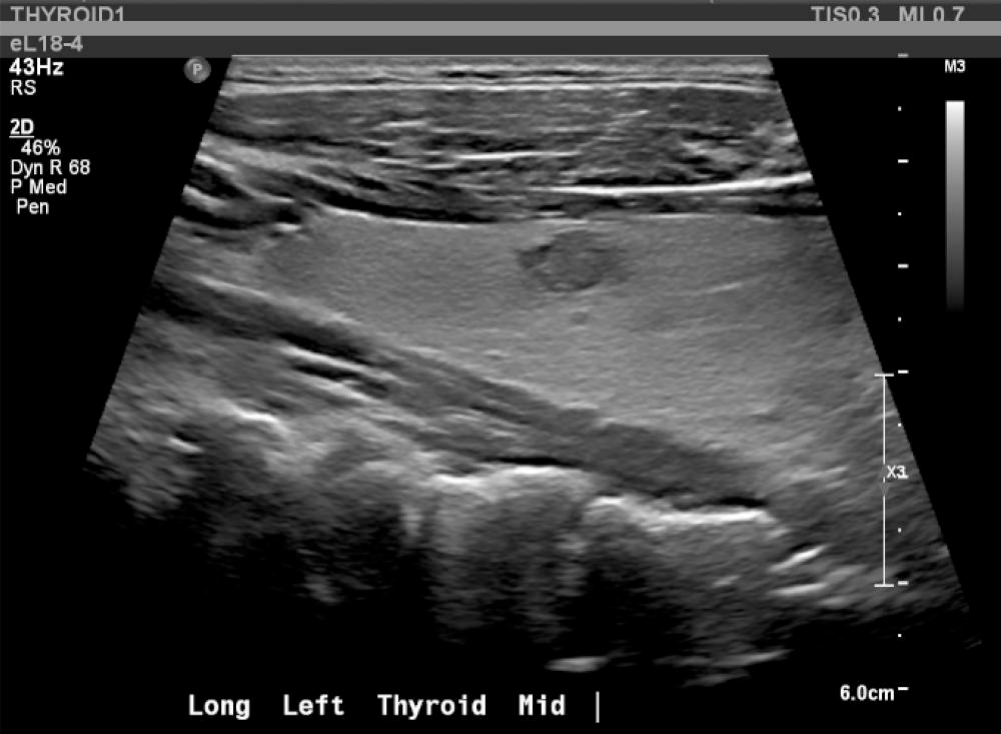
Left anterior mid gland: 10 × 6 × 10 mm.

**Figure 2.**
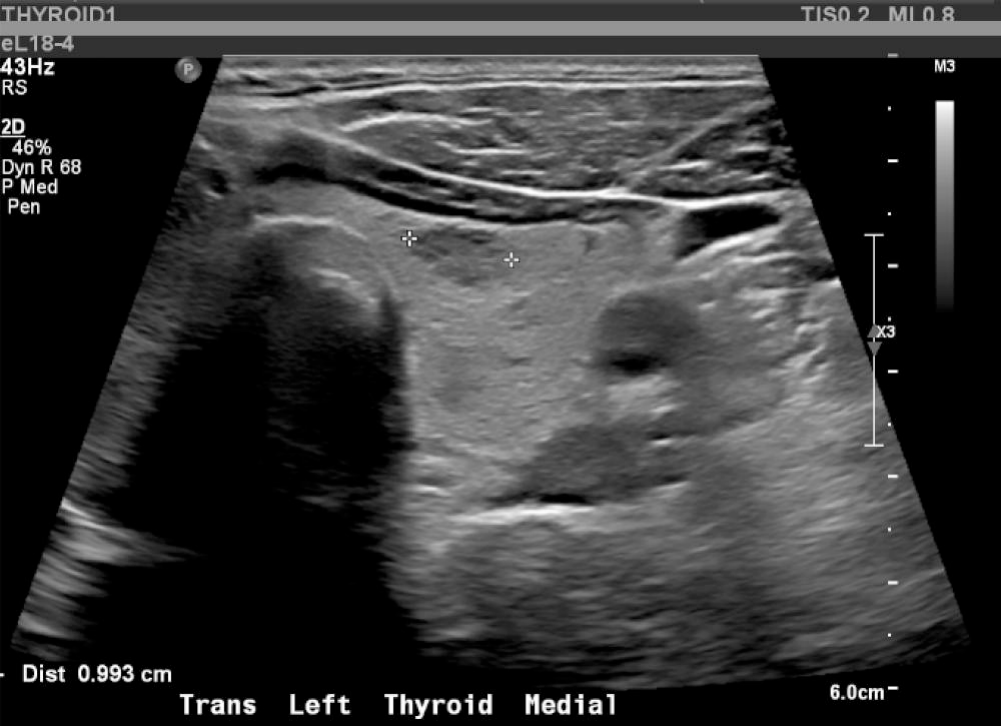
Left medial mid gland: 10 × 11 × 6 mm nodule.

**Figure 3.**
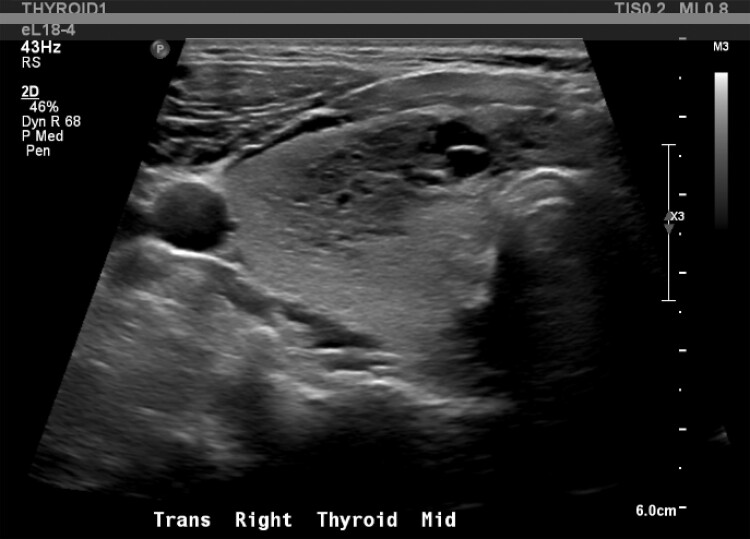
Right anterior mid gland: 36 × 18 × 33 mm spongiform nodule.

**Figure 4.**
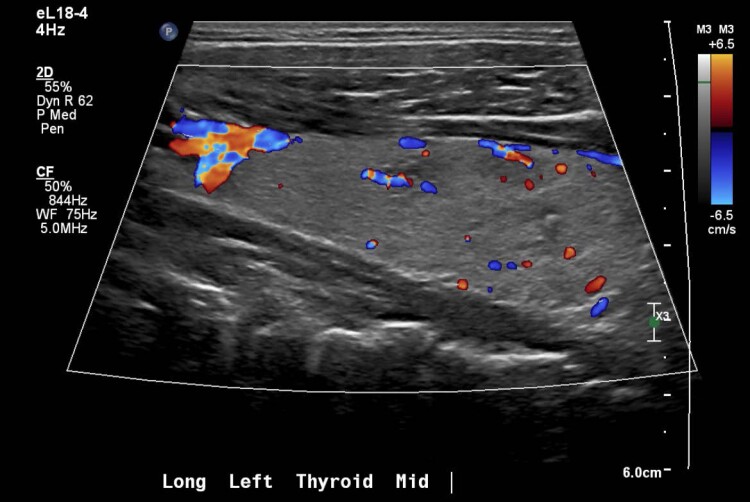
Longitudinal: left medial mid gland with Doppler.

**Figure 5.**
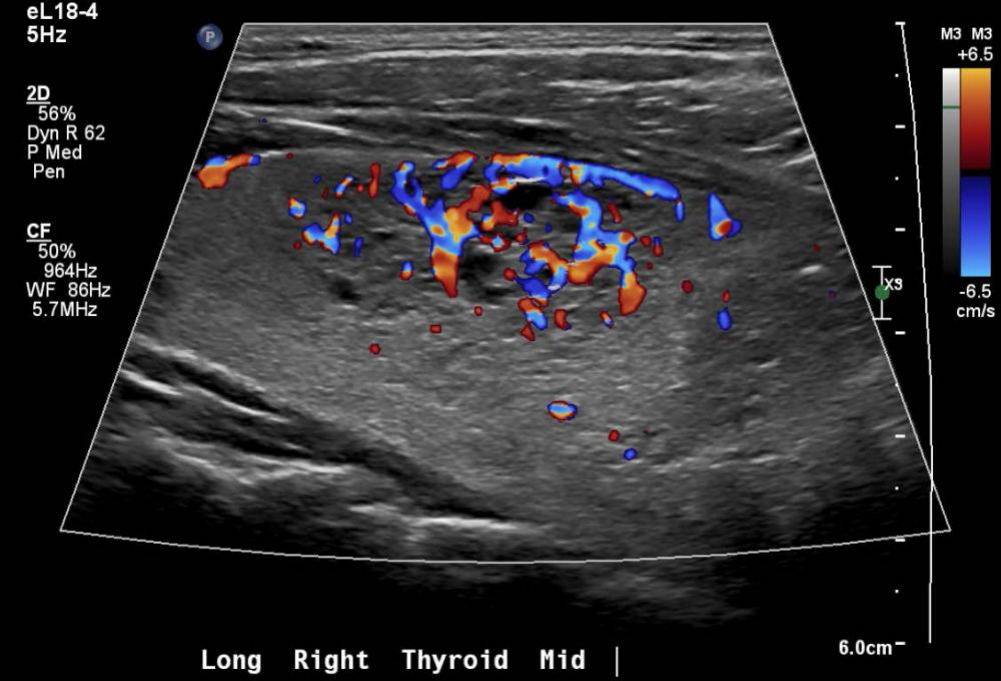
Longitudinal: right medial mid gland with Doppler.

**Figure 6.**
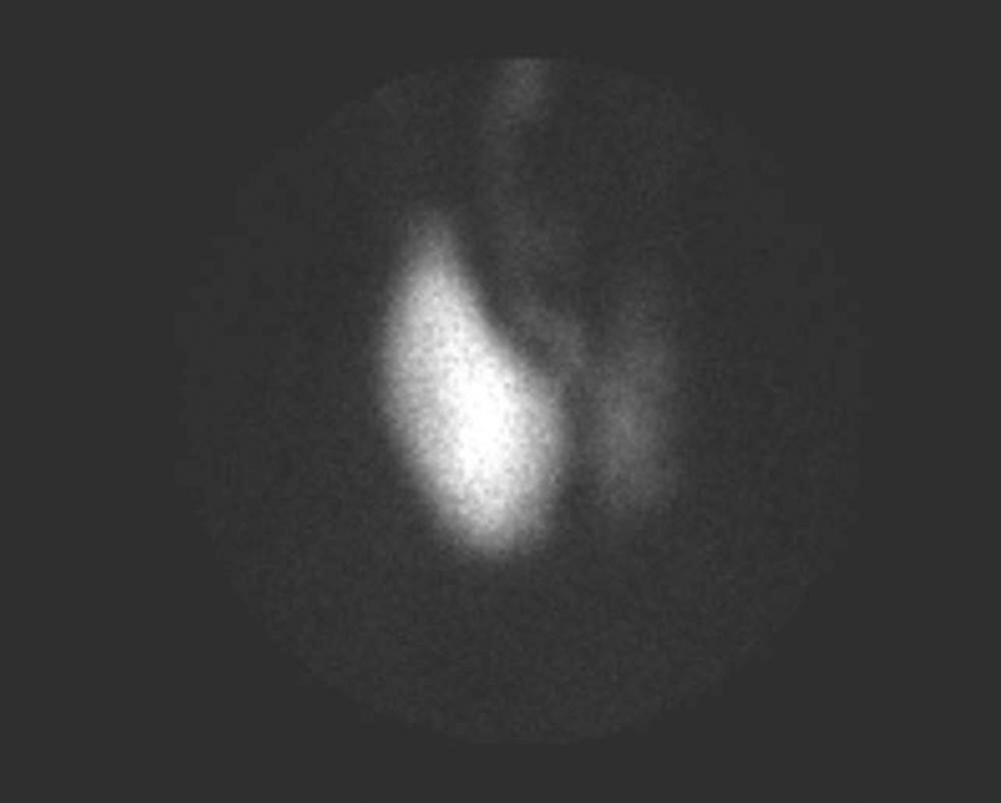
Tc-99m pertechnetate scan.

Biochemical evaluation showed mildly suppressed TSH 0.39 mIU/L (0.39 mcIU/mL; reference range, 0.47-4.68 mcIU/mL) but normal free T4, 13.41 pmol/L (1.04 ng/dL; reference range, 0.6-2.5 ng/dL) and total T3, 2.0 nmol/L (130 ng/dL; reference range, 97-169 ng/dL). Thyroid stimulating immunoglobulin (TSI) was elevated (467%; reference range, < 140% Basel) with normal thyroid receptor antibody (TRAb) (Table 1). A thyroid ultrasound demonstrated slightly enlarged left thyroid lobe with normal echogenicity (6.5 × 2.5 × 2.3 cm), 2 small left-sided nodules (10 × 11 × 6 mm and 10 × 6 × 10 mm), and right-sided thyromegaly (7.1 × 3.4 × 3.6 cm) with right benign spongiform nodule (36 × 18 × 33 mm) (Figs. 1-5). A nuclear medicine I-131 uptake and scan demonstrated unilateral right-sided uptake, with the left side demonstrating comparatively minimal uptake. One of the 2 nodules had a slight increase in uptake compared with the rest of the left side, but still much less than the entirety of the right side (Fig. 6).

**Table 1.** Thyroid function tests over time

	Reference range	1-y preoperation	3 wk preoperation	3 wk postoperation	4 mo postoperation	1 y postoperation
TSH	0.47-4.68 mcIU/mL	0.39	<0.02	3.36	2.27	2.21
Free T4	0.60-2.50 ng/dL	1.04	1.03	0.81	0.99	1.08
Total T3	97-169 ng/dL	130	—	—	120	117
TSI	<140% basal	467	—	—	332	—
Thyroid receptor antibody	≤2.00 IU/L	1.82	—	—	6.73	—

The patient underwent 2 left thyroid nodule fine needle aspiration biopsies to guide definitive therapy and consideration of partial vs total thyroidectomy. Biopsies demonstrated benign pathology.

## Treatment

Because of low TSH and development of symptoms of palpitations, insomnia, and dysphagia, the patient’s methimazole dose was escalated to 5 and 10 mg, alternating every other day. However, given his degree of elevated TSI, long-term remission was less likely. Therefore, definitive therapy was recommended. With underlying active Graves orbitopathy, radioactive iodine was relatively contraindicated, and total or partial thyroidectomy would be recommended.

## Outcome and Follow-up

Thyroid eye disease persisted, with continued proptosis, right-sided predominant. A right-sided hemithyroidectomy was performed because of strong patient preference against total thyroidectomy. Histopathology of the resected thyroid confirmed Graves disease. Methimazole was stopped at the time of surgery. Repeat biochemical evaluation 4 months postoperatively revealed normal TSH, normal free T4, and normal total T3 (Table 1). However, a new elevation in TRAb was noted, and TSI remained elevated (Table 1). The patient denied any thyrotoxic symptoms. On follow-up 1 year after the surgery, repeat biochemical testing of TSH, free T4, and total T3 remains within normal limits despite remaining off methimazole (Table 1).

## Discussion

The prevalence of hyperthyroidism is approximately 1.3% of the population, and Graves disease is the most common etiology [1]. Unilateral Graves disease is a rare presentation with approximately 20 cases reported worldwide [2–7]. Previously identified cases of unilateral Graves disease revealed both females and males being affected equally, despite Graves disease's preponderance for females in the general population [1,2]. The majority of the unilateral Graves disease cases in the literature involve right-sided uptake [3]. Response to treatment appears variable. Although some patients went into remission with methimazole, other cases required hemithyroidectomy. In 2 cases, methimazole had to be resumed after Graves disease recurred in the residual thyroid tissue after hemithyroidectomy [4].

The underlying pathophysiology of unilateral Graves disease is unclear. Hypotheses include differences in the amino acid structure of the thyroid antigens in each lobe that may result in asymmetrical B-cell infiltration in the thyroid, differences in TSH receptors, and differences in the sodium-iodine symporter expression between the thyroid lobes [2]. The presence of residual thyroid tissue (thyroglobulin level > 0.5 ng/dL) and Graves ophthalmopathy are strong predictors for persistently elevated autoantibody levels after surgery and may indicate a potential risk for relapse [8].

Of note, the patient we describe here had elevated TSI/TRAb titers following hemithyroidectomy surgery despite initial normal TRAb. A case report by Sakata et al presented 2 cases of unilateral involvement of the thyroid in Graves disease in which both patients had initial negative TRAb values. After undergoing hemithyroidectomy, they presented 27 months (case 1) and 6 months (case 2) later with relapse of symptoms, as well as biochemical testing suggestive of hyperthyroidism including newly positive TRAb levels and increased uptake of I123 in the retained thyroid lobe.

In summary, this report describes a rare presentation of Graves disease with unilateral thyroid lobe involvement. Given the rarity of this presentation, further research and understanding are needed to further elucidate the pathophysiology and help guide treatment decisions, especially with regard to the efficacy of total vs partial hemithyroidectomy in these cases.

## Learning Points

- Graves disease can rarely present in a single thyroid lobe.
- Treatment options may include total vs partial thyroidectomy if remission is not achieved with methimazole.
- A potential correlation between initial negative TRAb with positive TSI and the unilateral involvement of Graves disease needs further investigation.
- Given the rarity, further investigation is needed to determine the natural course of this form of Graves disease.

## Contributors

All authors made an individual contribution to authorship. M.M., R.R., and K.M. were involved in the diagnosis and management of this patient and in writing the manuscript. Y.A. was involved in the literature review, writing the manuscript, and submission. All authors contributed in writing the discussion, reviewed, and approved the final draft.

## Data Availability

Original data generated and analyzed during this study are included in this published article.

## Abbreviations

TRAb: thyroid receptor antibody
TSI: thyroid stimulating immunoglobulin
